# 1-(5-Hy­droxy-2,2,8,8-tetra­methyl-2*H*,8*H*-pyrano[2,3-*f*]chromen-6-yl)ethanone

**DOI:** 10.1107/S160053681204055X

**Published:** 2012-09-29

**Authors:** Sunayna Pawar, Adele Cheddie, Bernard Omondi, Neil Anthony Koorbanally

**Affiliations:** a School of Chemistry and Physics, University of KwaZulu-Natal, Private Bag X54001, Durban 4000, South Africa

## Abstract

In the title compound, C_18_H_20_O_4_,the pyran ring of the chromene unit adopts a half-chair conformation. An intra­molecular O—H⋯O hydrogen bond occurs. In the crystal, mol­ecules are linked along the *b* axis by C—H⋯O hydrogen bonds.

## Related literature

The title compound is a precursor in the synthesis of biologically active prenylated chalcones, see: Adler & Baldwin (2009 ▶); Lee & Li (2007 ▶); For related structures, see: Lee & Xia (2007 ▶); Mondal *et al.* (2007 ▶); Narender *et al.* (2005 ▶).
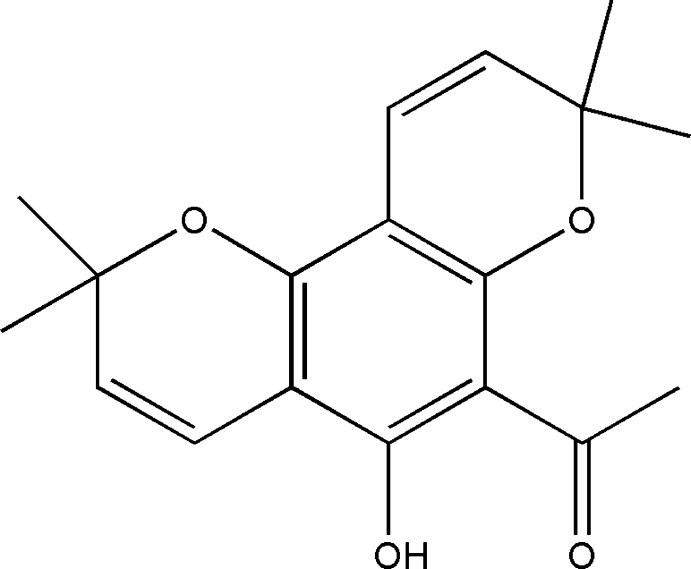


## Experimental

### 

#### Crystal data

C_18_H_20_O_4_
*M* *_r_* = 300.34
Triclinic, 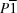

*a* = 8.5039 (6) Å
*b* = 9.5370 (6) Å
*c* = 10.7859 (7) Å
α = 102.180 (3)°
β = 102.621 (3)°
γ = 110.671 (3)°
*V* = 757.86 (9) Å^3^
*Z* = 2
Mo *K*α radiation
μ = 0.09 mm^−1^
*T* = 446 K
0.39 × 0.21 × 0.2 mm

#### Data collection

Bruker SMART APEXII CCD diffractometer
Absorption correction: multi-scan (*SADABS*; Bruker, 2008 ▶) *T*_min_ = 0.965, *T*_max_ = 0.982
17650 measured reflections
3770 independent reflections
3284 reflections with *I* > 2σ(*I*)
*R*_int_ = 0.018

#### Refinement

*R*[*F* ^2^ > 2σ(*F* ^2^)] = 0.039
*wR*(*F* ^2^) = 0.119
*S* = 1.06
3770 reflections
205 parameters
H-atom parameters constrained
Δρ_max_ = 0.37 e Å^−3^
Δρ_min_ = −0.27 e Å^−3^

### 

Data collection: *APEX2* (Bruker, 2008 ▶); cell refinement: *SAINT-Plus* (Bruker, 2008 ▶); data reduction: *SAINT-Plus* and *XPREP* (Bruker, 2008 ▶); program(s) used to solve structure: *SHELXS97* (Sheldrick, 2008 ▶); program(s) used to refine structure: *SHELXL97* (Sheldrick, 2008 ▶); molecular graphics: *ORTEP-3* (Farrugia, 2012 ▶); software used to prepare material for publication: *WinGX* (Farrugia, 2012 ▶).

## Supplementary Material

Crystal structure: contains datablock(s) global, I. DOI: 10.1107/S160053681204055X/hg5251sup1.cif

Structure factors: contains datablock(s) I. DOI: 10.1107/S160053681204055X/hg5251Isup2.hkl

Additional supplementary materials:  crystallographic information; 3D view; checkCIF report

## Figures and Tables

**Table 1:** Hydrogen-bond geometry (Å, °)

*D*—H⋯*A*	*D*—H	H⋯*A*	*D*⋯*A*	*D*—H⋯*A*
O3—H3⋯O2	0.82	1.76	2.4897 (11)	148
C11—H11*A*⋯O2^i^	0.96	2.48	3.3829 (14)	156

Symmetry code: (i) 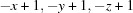.

**Fig. 1.**
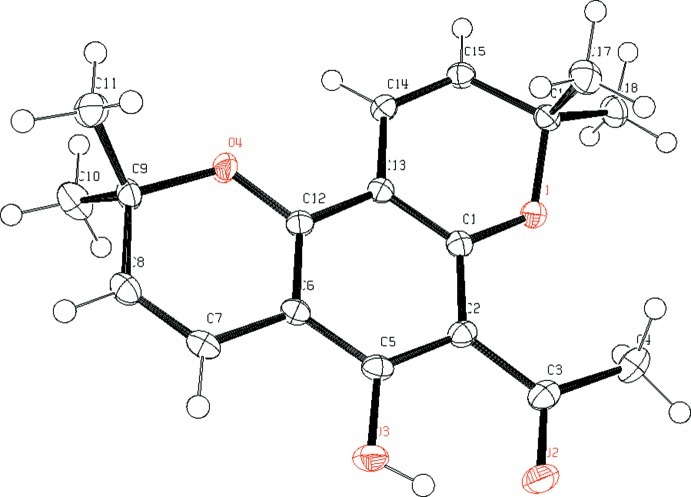
ORTEP diagram showing the molecular structure of the titled compound with atomic labelling scheme. Non-H atoms are drawn with 50% probability displacement ellipsoids and H atoms are shown as open circles.

## supplementary crystallographic information

### Comment

The title compound (I), a pyranochromene acetophenone, was obtained as an intermediate in the synthesis of biologically active chalcones and flavanones (Adler & Baldwin, 2009; Lee & Li 2007; Lee & Xia, 2007). The pyranochromene skeleton is a core structure in various naturally active compounds (Adler and Baldwin 2009, Narender *et al.* 2005, Mondal *et al.* 2007). The efficient and concise synthesis of pyranochalcones was achieved from readily available 2,4,6-trihydroxyacetophenone. The key step in the synthetic strategy was a base catalyzed benzopyran formation. The crystal structure of the title compound (Fig. 1) has not been previously reported. One of the pyran rings of the chromene unit forms a half chair conformation [Q = 0.3846 (10) Å, θ = 112.81 (16)° and ψ = 142.65 (17) °]. The maximum displacement from the C1O1C13C14 plane are 0.684 Å for O16 and 0.275 Å for C15. In the crystal structure of the title compound, molecules are linked together by a C—H···O hydrogen bond along the crystallographic *b* axis (Table 1).

### Experimental

To a one necked round bottom flask, 2,4,6-trihydroxyacetophenone (2.0 g, 0.0119 mol) and 2,3-dimethylbutenal (4.0 g, 0.0476 mol) was added. This was followed by the addition of pyridine (1.35 g) and the reaction mixture stirred for 24 h at 110°C. The reaction was monitored by TLC using EtOAc: Hexane (5:95, *R*_f_ = 0.6). After completion of the reaction, hydrochloric acid (30 ml) was added to neutralize the reaction and the mixture extracted with ethyl acetate (4 *x* 40 ml). The combined organic layer was dried over MgSO_4_ and the solvent evaporated under reduced pressure. The residue was purified by column chromatography on silica gel using 100% hexane as the eluent to afford the pyranochromene as a yellow crystalline solid (2.58 g, yield 72.82%) with a melting point of 89–90 °C.

Recrystallization from hexane at room temperature afforded yellow crystals suitable for X-ray analysis.

^1^H NMR (400 MHz, CDCl_3_): *δ* (p.p.m.): 13.99 (1*H*, s, OH), 6.65 (1*H*, d, *J* = 10.08 Hz), 6.58 (1*H*, d, *J* = 10.08 Hz), 5.45 (1*H*, d, *J* = 10.08 Hz), 5.43 (1*H*, d, *J* = 10.08 Hz), 2.65 (3*H*, s), 1.49 (6*H*, s), 1.43 (6*H*, s).

^13^C NMR (100 MHz, CDCl_3_): *δ* (p.p.m.): 203.2, 160.5, 156.6, 154.9, 125.3, 124.6, 116.4, 116.1, 105.7, 102.2, 102.1, 78.2, 78.1, 33.1, 28.5, 28.2.

### Refinement

Carbon-bound H-atoms were placed in calculated positions [C—H = 0.96 Å for Me H atoms and 0.93 Å for aromatic H atoms; *U*_iso_(H) = 1.2*U*_eq_(C) (1.5 for Me groups)] and were included in the refinement in the riding model approximation. The O—H H-atom was located in a difference map and also placed in a calculated position O—H = 0.82 Å (*U*_iso_(H) = 1.2*U*_eq_(O).

### Crystal data

**Table:**

C_18_H_20_O_4_	*Z* = 2
*M**_r_* = 300.34	*F*(000) = 320
Triclinic, *P*1	*D*_x_ = 1.316 Mg m^−^^3^
Hall symbol: -P 1	Mo *K*α radiation, λ = 0.71073 Å
*a* = 8.5039 (6) Å	Cell parameters from 17650 reflections
*b* = 9.5370 (6) Å	θ = 2.0–28.5°
*c* = 10.7859 (7) Å	µ = 0.09 mm^−^^1^
α = 102.180 (3)°	*T* = 446 K
β = 102.621 (3)°	Block, yellow
γ = 110.671 (3)°	0.39 × 0.21 × 0.2 mm
*V* = 757.86 (9) Å^3^	

### Data collection

**Table:**

Bruker SMART APEXII CCD diffractometer	3284 reflections with *I* > 2σ(*I*)
Graphite monochromator	*R*_int_ = 0.018
φ and ω scans	θ_max_ = 28.5°, θ_min_ = 2.0°
Absorption correction: multi-scan (*SADABS*; Bruker, 2008)	*h* = −11→10
*T*_min_ = 0.965, *T*_max_ = 0.982	*k* = −12→12
17650 measured reflections	*l* = −14→14
3770 independent reflections	

### Refinement

**Table:**

Refinement on *F*^2^	Primary atom site location: structure-invariant direct methods
Least-squares matrix: full	Secondary atom site location: difference Fourier map
*R*[*F*^2^ > 2σ(*F*^2^)] = 0.039	Hydrogen site location: inferred from neighbouring sites
*wR*(*F*^2^) = 0.119	H-atom parameters constrained
*S* = 1.06	*w* = 1/[σ^2^(*F*_o_^2^) + (0.0701*P*)^2^ + 0.1777*P*] where *P* = (*F*_o_^2^ + 2*F*_c_^2^)/3
3770 reflections	(Δ/σ)_max_ = 0.001
205 parameters	Δρ_max_ = 0.37 e Å^−^^3^
0 restraints	Δρ_min_ = −0.27 e Å^−^^3^

### Special details

**Table:**

Experimental. Carbon-bound H-atoms were placed in calculated positions [C—H = 0.96 Å for Me H atoms and 0.93 Å for aromatic H atoms; *U*_iso_(H) = 1.2*U*_eq_(C) (1.5 for Me groups)] and were included in the refinement in the riding model approximation. The O—H H-atom was located in a difference map and also placed in a calculated position O—H = 0.82 Å (*U*_iso_(H) = 1.2*U*_eq_(O).
Geometry. All e.s.d.'s (except the e.s.d. in the dihedral angle between two l.s. planes) are estimated using the full covariance matrix. The cell e.s.d.'s are taken into account individually in the estimation of e.s.d.'s in distances, angles and torsion angles; correlations between e.s.d.'s in cell parameters are only used when they are defined by crystal symmetry. An approximate (isotropic) treatment of cell e.s.d.'s is used for estimating e.s.d.'s involving l.s. planes.
Refinement. Refinement of *F*^2^ against ALL reflections. The weighted *R*-factor *wR* and goodness of fit *S* are based on *F*^2^, conventional *R*-factors *R* are based on *F*, with *F* set to zero for negative *F*^2^. The threshold expression of *F*^2^ > σ(*F*^2^) is used only for calculating *R*-factors(gt) *etc*. and is not relevant to the choice of reflections for refinement. *R*-factors based on *F*^2^ are statistically about twice as large as those based on *F*, and *R*- factors based on ALL data will be even larger. >>> The Following Model ALERTS were generated - (Acta-Mode) <<< Format: alert-number_ALERT_alert-type_alert-level text 911_ALERT_3_C Missing # FCF Refl Between THmin & STh/*L*= 0.600 5 912_ALERT_4_C Missing # of FCF Reflections Above STh/*L*= 0.600 62 154_ALERT_1_G The su's on the Cell Angles are Equal (*x* 10000) 300 Deg. Noted:

### Fractional atomic coordinates and isotropic or equivalent isotropic displacement parameters (Å^2^)

**Table:**

	*x*	*y*	*z*	*U*_iso_*/*U*_eq_	
C1	0.19888 (12)	0.60398 (11)	0.46668 (9)	0.01525 (19)	
C2	0.20857 (12)	0.45580 (11)	0.43556 (9)	0.0169 (2)	
C3	0.12126 (13)	0.33380 (11)	0.30439 (10)	0.0199 (2)	
C4	0.01442 (14)	0.35565 (13)	0.18500 (10)	0.0252 (2)	
H4A	−0.0205	0.2669	0.1072	0.038*	
H4B	0.085	0.4506	0.1705	0.038*	
H4C	−0.0894	0.3633	0.201	0.038*	
C5	0.31333 (13)	0.42621 (11)	0.54112 (10)	0.0170 (2)	
C6	0.40358 (12)	0.53801 (11)	0.66846 (9)	0.01595 (19)	
C7	0.51498 (13)	0.51113 (11)	0.77598 (10)	0.0190 (2)	
H7	0.5314	0.4184	0.7601	0.023*	
C8	0.59305 (13)	0.61881 (12)	0.89653 (10)	0.0205 (2)	
H8	0.6653	0.5995	0.9627	0.025*	
C9	0.57082 (13)	0.77035 (12)	0.93170 (9)	0.0183 (2)	
C10	0.47362 (16)	0.77313 (14)	1.03468 (11)	0.0279 (2)	
H10A	0.4601	0.8703	1.0553	0.042*	
H10B	0.5408	0.765	1.1147	0.042*	
H10C	0.3588	0.6858	0.9989	0.042*	
C11	0.74885 (14)	0.91349 (12)	0.98215 (11)	0.0250 (2)	
H11A	0.8052	0.9128	0.9141	0.037*	
H11B	0.8232	0.9096	1.0612	0.037*	
H11C	0.7304	1.0083	1.003	0.037*	
C12	0.38550 (12)	0.68003 (11)	0.69361 (9)	0.01523 (19)	
C13	0.28325 (12)	0.71568 (11)	0.59435 (9)	0.01472 (19)	
C14	0.25158 (12)	0.85820 (11)	0.61994 (9)	0.01643 (19)	
H14	0.2817	0.9215	0.7074	0.02*	
C15	0.17926 (12)	0.89745 (11)	0.51758 (9)	0.0176 (2)	
H15	0.1525	0.9848	0.5333	0.021*	
C16	0.14111 (12)	0.79960 (11)	0.37625 (9)	0.0174 (2)	
C17	0.30306 (14)	0.85631 (13)	0.32954 (10)	0.0240 (2)	
H17A	0.4006	0.8484	0.3875	0.036*	
H17B	0.3348	0.9643	0.3322	0.036*	
H17C	0.2755	0.7919	0.2396	0.036*	
C18	−0.02224 (13)	0.79449 (13)	0.27938 (10)	0.0229 (2)	
H18A	−0.0464	0.7254	0.192	0.034*	
H18B	−0.001	0.8988	0.2749	0.034*	
H18C	−0.1222	0.756	0.3101	0.034*	
O1	0.09524 (9)	0.63381 (8)	0.36994 (7)	0.01782 (16)	
O2	0.13363 (11)	0.20500 (9)	0.28795 (8)	0.02575 (18)	
O3	0.33104 (10)	0.28943 (8)	0.52254 (7)	0.02275 (18)	
H3	0.2748	0.2324	0.4451	0.034*	
O4	0.46499 (10)	0.79211 (8)	0.81538 (7)	0.02102 (17)	

### Atomic displacement parameters (Å^2^)

**Table:**

	*U*^11^	*U*^22^	*U*^33^	*U*^12^	*U*^13^	*U*^23^
C1	0.0132 (4)	0.0163 (4)	0.0162 (4)	0.0051 (3)	0.0054 (3)	0.0060 (3)
C2	0.0165 (4)	0.0147 (4)	0.0177 (4)	0.0042 (3)	0.0069 (3)	0.0039 (3)
C3	0.0171 (4)	0.0171 (4)	0.0214 (5)	0.0026 (4)	0.0089 (4)	0.0031 (4)
C4	0.0213 (5)	0.0244 (5)	0.0200 (5)	0.0060 (4)	0.0026 (4)	−0.0015 (4)
C5	0.0182 (4)	0.0140 (4)	0.0212 (5)	0.0065 (3)	0.0100 (4)	0.0069 (3)
C6	0.0156 (4)	0.0153 (4)	0.0189 (4)	0.0066 (3)	0.0072 (3)	0.0074 (4)
C7	0.0198 (4)	0.0179 (4)	0.0248 (5)	0.0106 (4)	0.0088 (4)	0.0110 (4)
C8	0.0203 (5)	0.0228 (5)	0.0225 (5)	0.0120 (4)	0.0054 (4)	0.0111 (4)
C9	0.0197 (4)	0.0209 (5)	0.0152 (4)	0.0104 (4)	0.0029 (3)	0.0072 (3)
C10	0.0350 (6)	0.0347 (6)	0.0284 (5)	0.0224 (5)	0.0167 (5)	0.0173 (5)
C11	0.0208 (5)	0.0225 (5)	0.0265 (5)	0.0087 (4)	0.0005 (4)	0.0058 (4)
C12	0.0139 (4)	0.0146 (4)	0.0164 (4)	0.0051 (3)	0.0050 (3)	0.0048 (3)
C13	0.0142 (4)	0.0148 (4)	0.0158 (4)	0.0061 (3)	0.0053 (3)	0.0054 (3)
C14	0.0160 (4)	0.0161 (4)	0.0162 (4)	0.0069 (3)	0.0045 (3)	0.0035 (3)
C15	0.0172 (4)	0.0164 (4)	0.0199 (4)	0.0081 (3)	0.0057 (3)	0.0058 (3)
C16	0.0182 (4)	0.0168 (4)	0.0175 (4)	0.0072 (3)	0.0050 (3)	0.0071 (3)
C17	0.0223 (5)	0.0264 (5)	0.0240 (5)	0.0078 (4)	0.0108 (4)	0.0103 (4)
C18	0.0218 (5)	0.0255 (5)	0.0201 (5)	0.0098 (4)	0.0023 (4)	0.0094 (4)
O1	0.0179 (3)	0.0168 (3)	0.0161 (3)	0.0064 (3)	0.0022 (3)	0.0048 (3)
O2	0.0313 (4)	0.0160 (3)	0.0258 (4)	0.0065 (3)	0.0112 (3)	0.0023 (3)
O3	0.0300 (4)	0.0151 (3)	0.0251 (4)	0.0111 (3)	0.0101 (3)	0.0059 (3)
O4	0.0261 (4)	0.0184 (3)	0.0152 (3)	0.0121 (3)	−0.0017 (3)	0.0023 (3)

### Geometric parameters (Å, º)

**Table:**

C1—O1	1.3590 (11)	C10—H10B	0.96
C1—C13	1.3984 (13)	C10—H10C	0.96
C1—C2	1.4180 (13)	C11—H11A	0.96
C2—C5	1.4281 (13)	C11—H11B	0.96
C2—C3	1.4640 (13)	C11—H11C	0.96
C3—O2	1.2485 (12)	C12—O4	1.3543 (11)
C3—C4	1.5042 (14)	C12—C13	1.4048 (12)
C4—H4A	0.96	C13—C14	1.4590 (12)
C4—H4B	0.96	C14—C15	1.3332 (13)
C4—H4C	0.96	C14—H14	0.93
C5—O3	1.3439 (11)	C15—C16	1.5065 (13)
C5—C6	1.3986 (13)	C15—H15	0.93
C6—C12	1.3935 (12)	C16—O1	1.4711 (11)
C6—C7	1.4577 (13)	C16—C18	1.5232 (13)
C7—C8	1.3282 (14)	C16—C17	1.5281 (13)
C7—H7	0.93	C17—H17A	0.96
C8—C9	1.5038 (13)	C17—H17B	0.96
C8—H8	0.93	C17—H17C	0.96
C9—O4	1.4749 (11)	C18—H18A	0.96
C9—C11	1.5228 (14)	C18—H18B	0.96
C9—C10	1.5238 (14)	C18—H18C	0.96
C10—H10A	0.96	O3—H3	0.82
			
O1—C1—C13	119.17 (8)	C9—C11—H11A	109.5
O1—C1—C2	118.15 (8)	C9—C11—H11B	109.5
C13—C1—C2	122.58 (8)	H11A—C11—H11B	109.5
C1—C2—C5	116.61 (8)	C9—C11—H11C	109.5
C1—C2—C3	124.92 (9)	H11A—C11—H11C	109.5
C5—C2—C3	118.47 (9)	H11B—C11—H11C	109.5
O2—C3—C2	120.10 (9)	O4—C12—C6	122.09 (8)
O2—C3—C4	117.13 (9)	O4—C12—C13	115.82 (8)
C2—C3—C4	122.76 (9)	C6—C12—C13	122.09 (8)
C3—C4—H4A	109.5	C1—C13—C12	118.05 (8)
C3—C4—H4B	109.5	C1—C13—C14	118.47 (8)
H4A—C4—H4B	109.5	C12—C13—C14	123.36 (8)
C3—C4—H4C	109.5	C15—C14—C13	119.82 (8)
H4A—C4—H4C	109.5	C15—C14—H14	120.1
H4B—C4—H4C	109.5	C13—C14—H14	120.1
O3—C5—C6	116.62 (9)	C14—C15—C16	119.64 (8)
O3—C5—C2	121.48 (9)	C14—C15—H15	120.2
C6—C5—C2	121.89 (9)	C16—C15—H15	120.2
C12—C6—C5	118.72 (8)	O1—C16—C15	109.49 (7)
C12—C6—C7	118.82 (8)	O1—C16—C18	104.23 (7)
C5—C6—C7	122.46 (9)	C15—C16—C18	112.21 (8)
C8—C7—C6	120.13 (9)	O1—C16—C17	108.08 (8)
C8—C7—H7	119.9	C15—C16—C17	111.13 (8)
C6—C7—H7	119.9	C18—C16—C17	111.38 (8)
C7—C8—C9	123.48 (9)	C16—C17—H17A	109.5
C7—C8—H8	118.3	C16—C17—H17B	109.5
C9—C8—H8	118.3	H17A—C17—H17B	109.5
O4—C9—C8	112.70 (8)	C16—C17—H17C	109.5
O4—C9—C11	104.96 (8)	H17A—C17—H17C	109.5
C8—C9—C11	111.35 (8)	H17B—C17—H17C	109.5
O4—C9—C10	106.15 (8)	C16—C18—H18A	109.5
C8—C9—C10	110.78 (8)	C16—C18—H18B	109.5
C11—C9—C10	110.66 (9)	H18A—C18—H18B	109.5
C9—C10—H10A	109.5	C16—C18—H18C	109.5
C9—C10—H10B	109.5	H18A—C18—H18C	109.5
H10A—C10—H10B	109.5	H18B—C18—H18C	109.5
C9—C10—H10C	109.5	C1—O1—C16	117.84 (7)
H10A—C10—H10C	109.5	C5—O3—H3	109.5
H10B—C10—H10C	109.5	C12—O4—C9	122.57 (7)
			
O1—C1—C2—C5	−177.70 (8)	C7—C6—C12—C13	178.75 (8)
C13—C1—C2—C5	−1.41 (14)	O1—C1—C13—C12	178.20 (8)
O1—C1—C2—C3	2.90 (14)	C2—C1—C13—C12	1.95 (14)
C13—C1—C2—C3	179.19 (8)	O1—C1—C13—C14	2.07 (13)
C1—C2—C3—O2	−177.92 (9)	C2—C1—C13—C14	−174.19 (8)
C5—C2—C3—O2	2.69 (14)	O4—C12—C13—C1	179.79 (8)
C1—C2—C3—C4	2.05 (15)	C6—C12—C13—C1	−0.41 (14)
C5—C2—C3—C4	−177.34 (8)	O4—C12—C13—C14	−4.28 (13)
C1—C2—C5—O3	179.80 (8)	C6—C12—C13—C14	175.53 (8)
C3—C2—C5—O3	−0.76 (14)	C1—C13—C14—C15	−15.41 (13)
C1—C2—C5—C6	−0.67 (14)	C12—C13—C14—C15	168.67 (9)
C3—C2—C5—C6	178.77 (8)	C13—C14—C15—C16	−3.69 (13)
O3—C5—C6—C12	−178.33 (8)	C14—C15—C16—O1	32.30 (12)
C2—C5—C6—C12	2.12 (14)	C14—C15—C16—C18	147.53 (9)
O3—C5—C6—C7	1.33 (14)	C14—C15—C16—C17	−87.03 (11)
C2—C5—C6—C7	−178.22 (8)	C13—C1—O1—C16	29.94 (12)
C12—C6—C7—C8	1.98 (14)	C2—C1—O1—C16	−153.63 (8)
C5—C6—C7—C8	−177.68 (9)	C15—C16—O1—C1	−45.75 (10)
C6—C7—C8—C9	1.28 (16)	C18—C16—O1—C1	−165.98 (8)
C7—C8—C9—O4	−4.60 (14)	C17—C16—O1—C1	75.44 (10)
C7—C8—C9—C11	−122.24 (11)	C6—C12—O4—C9	−2.36 (14)
C7—C8—C9—C10	114.16 (11)	C13—C12—O4—C9	177.44 (8)
C5—C6—C12—O4	178.22 (8)	C8—C9—O4—C12	5.12 (13)
C7—C6—C12—O4	−1.45 (14)	C11—C9—O4—C12	126.46 (9)
C5—C6—C12—C13	−1.58 (14)	C10—C9—O4—C12	−116.31 (10)

### Hydrogen-bond geometry (Å, º)

**Table:**

*D*—H···*A*	*D*—H	H···*A*	*D*···*A*	*D*—H···*A*
O3—H3···O2	0.82	1.76	2.4897 (11)	148
C11—H11*A*···O2^i^	0.96	2.48	3.3829 (14)	156

Symmetry code: (i) −*x*+1, −*y*+1, −*z*+1.
